# Specific delivery of microRNA93 into HBV-replicating hepatocytes downregulates protein expression of liver cancer susceptible gene MICA

**DOI:** 10.18632/oncotarget.2143

**Published:** 2014-06-26

**Authors:** Motoko Ohno, Motoyuki Otsuka, Takahiro Kishikawa, Chikako Shibata, Takeshi Yoshikawa, Akemi Takata, Ryosuke Muroyama, Norie Kowatari, Masaya Sato, Naoya Kato, Shun'ichi Kuroda, Kazuhiko Koike

**Affiliations:** ^1^ Department of Gastroenterology, Graduate School of Medicine, The University of Tokyo, Tokyo, Japan; ^2^ Japan Science and Technology Agency, PRESTO, Kawaguchi, Saitama, Japan; ^3^ Unit of Disease Control Genome Medicine, Institute of Medical Science, The University of Tokyo, Tokyo, Japan; ^4^ Graduate School of Bioagricultural Sciences, Nagoya University, Nagoya, Japan

**Keywords:** Hepatitis, Bionanocapsules, Drug delivery, Primary hepatocyte

## Abstract

Chronic hepatitis B virus (HBV) infection is a major cause of hepatocellular carcinoma (HCC). To date, the lack of efficient in vitro systems supporting HBV infection and replication has been a major limitation of HBV research. Although primary human hepatocytes support the complete HBV life cycle, their limited availability and difficulties with gene transduction remain problematic. Here, we used human primary hepatocytes isolated from humanized chimeric uPA/SCID mice as efficient sources. These hepatocytes supported HBV replication in vitro. Based on analyses of mRNA and microRNA (miRNA) expression levels in HBV-infected hepatocytes, miRNA93 was significantly downregulated during HBV infection. MiRNA93 is critical for regulating the expression levels of MICA protein, which is a determinant for HBV-induced HCC susceptibility. Exogenous addition of miRNA93 in HBV-infected hepatocytes using bionanocapsules consisted of HBV envelope L proteins restored MICA protein expression levels in the supernatant. These results suggest that the rescued suppression of soluble MICA protein levels by miRNA93 targeted to HBV-infected hepatocytes using bionanocapsules may be useful for the prevention of HBV-induced HCC by altering deregulated miRNA93 expression.

## INTRODUCTION

Hepatitis B virus (HBV) infection is a major global health problem, and more than 350 million people globally are chronic carriers of the virus [1]. A significant number of these carriers suffer from either liver failure or hepatocellular carcinoma (HCC) during the late stages of the disease [2]. In fact, chronic infection with HBV is responsible for 60% of HCC cases in Asia and Africa and at least 20% those in Europe, Japan, and the United States [3].

While nucleoside and nucleotide analogs have been applied in the attempts to suppress HBV replication [4, 5], complete elimination of HBV (including cccDNA) remains difficult [6, 7], and an increased understanding of HBV replication and pathogenesis at the molecular level is essential for clinical management of chronic HBV infection. However, the lack of appropriate cell culture systems supporting stable and efficient HBV infection has been a major limitation. Although transient transfection or viral transfer of HBV genes or genomes are used in the study of specific steps of the HBV cell cycle [8-12], they do not accurately reflect the biology of HBV infection and replication. Thus, humanized mice are used for hepatitis virus research [13-18]. Although these mice are useful, immune deficient, chimeric mice are difficult to handle and maintain. Therefore, a more convenient *in vitro* system is required for HBV research.

Primary human hepatocytes can support the complete HBV life cycle *in vitro* [7, 19], but a major drawback is their limited availability. To overcome difficulties regarding availability, we used chimeric mice as sources of primary human hepatocytes, which grow robustly during the establishment of chimeric mice, due to continual liver damage induced by urokinase-type plasminogen activator (uPA) [14, 15].

Another shortcoming of utilizing primary human hepatocytes is their difficulty with gene transduction due to the low transfection efficiency of their primary cell-like nature. Efficient gene delivery methods will significantly improve studies on primary hepatocytes for HBV replication. In addition, cell-specific targeting is required for efficient drug delivery *in vivo*. As a specific gene delivery method to liver-derived cells, bionanocapsules (BNCs) consisted of HBV envelope L particles have been tested for the selective delivery of genes, drugs, or siRNAs into liver-derived cells [20, 21]. Because these BNCs are consisted of HBV L protein, they may be applicable for drug delivery to HBV-infected primary human hepatocytes.

MicroRNAs (miRNAs) are endogenous ~22-nucleotide RNAs that mediate important gene-regulatory events by base-pairing with mRNAs and activating their repression [22]. We previously reported that modifying the expression of miRNAs in liver cells can efficiently regulate the expression levels of the MHC class I polypeptide-related sequence A (MICA) protein [23], which we previously identified as a crucial factor for the susceptibility of hepatitis virus-induced HCC and possibly hepatitis virus clearance [24, 25]. While emerging evidence suggests that miRNAs play crucial roles in chronic HBV infection [26], the comprehensive changes in miRNA expression levels induced by HBV infection in human hepatocytes or in alternative systems reflecting HBV-infected hepatocytes have not been explored.

In this study, we infected primary human hepatocytes isolated from chimeric mice with HBV and identified the transcripts and miRNAs whose expression levels changed. We explored whether BNCs carrying synthesized miRNAs could successfully deliver miRNAs into primary hepatocytes and rescue the modulated miRNA expression due to HBV replication. We found that BNCs carrying synthesized miRNA93 could efficiently restore deregulated soluble MICA protein levels in the supernatant of HBV-replicating primary hepatocytes. These results suggest that miRNA93 delivery into HBV-replicating hepatocytes using BNC methods may enhance HBV immune clearance or suppress HCC by altering miRNA93 levels in HBV-infected cells.

## RESULTS

### Changes in expression levels of transcripts and miRNAs during HBV replication in human primary hepatocytes

**Figure 1:**
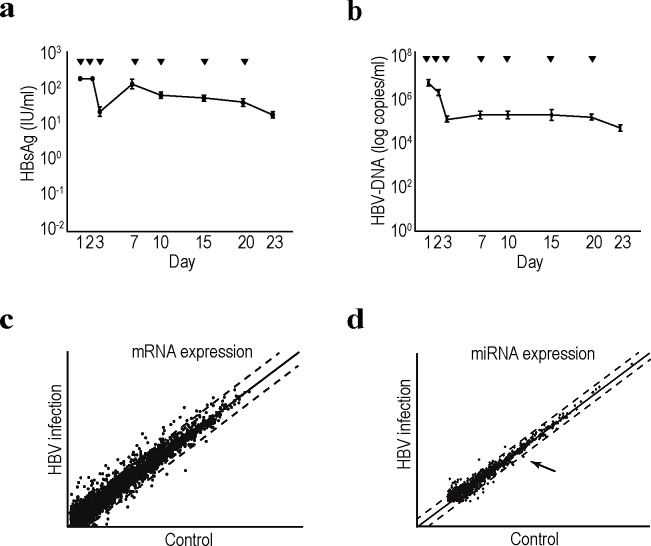
Comprehensive transcriptome and miRNA analyses in HBV-replicating human primary hepatocytes a, b, Efficient HBV replication in human primary hepatocytes isolated from chimeric mice. Primary human hepatocytes isolated from chimeric mice were seeded into the wells of a 24-well plate. Serum from HBV-infected patients was added to infect the cells with HBV. Media was changed at the indicated days (▼). The supernatant was collected when the media was changed for the analyses of HBsAg levels (a) and HBV-DNA levels (b). Data represent the means ± s.d. of three independent experiments. c, Scatter plot reflecting the transcriptomic results comparing the control and HBV-replicating primary human hepatocytes. Cells at day 7 after HBV infection were used for the analyses. Intensity normalization was performed using global normalization based on the expression levels of all genes analyzed. Dashed lines indicate the thresholds: two-fold increase or 50% decrease in expression levels. The full data are deposited in NCBI GEO database accession: GSE55928. d, A scatter plot of the miRNA microarray results was used to determine the expression levels of comprehensive mature miRNAs. Total RNA from control and HBV-replicating primary hepatocytes at day 7 after infection was used. Dashed lines indicate the thresholds: two-fold increase or 50% decrease in expression levels. Intensity normalization was performed using global normalization based on the expression levels of all miRNAs. The arrow indicates the result for miRNA93. The full data are deposited in NCBI GEO database accession: GSE55929.

We examined changes in transcript and miRNA expression levels during HBV infection and replication in hepatocytes. Primary human hepatocytes were used for maintainng HBV replication *in vitro*. We first isolated primary hepatocytes from humanized chimeric mice. To examine the infectivity of HBV into the primary hepatocytes *in vitro*, HBsAg and HBV-DNA levels in the cell culture supernatant were measured after the cells were infected with approximately 1.5 × 10^7^ copies of HBV/well in a 24-well plate at day 0. Although both HBsAg and HBV-DNA levels transiently decreased at approximately day 3, levels of both started to increase and were maintained until after day 23 post-infection (Figure 1a and b). These results suggested that human primary hepatocytes isolated from chimeric mice can efficiently support HBV replication *in vitro*, which can be used as an efficient *in vitro* HBV replication system.

To examine comprehensive changes in mRNA and miRNA expression levels in HBV-infected hepatocytes, cells at day 7 post-infection were collected and subjected to cDNA as well as miRNA microarrays. Among 24,460 genes examined, 65 were significantly upregulated by more than 4-fold, and 29 were downregulated to less than 25% (Supplementary Table 1 and 2); however, more than 800 total genes were upregulated or downregulated if the thresholds of the changes were set at 2-fold and 1.5-fold, respectively (Figure 1c; complete datasets have been deposited as GEO accession number: GSE55928). Among the upregulated genes, those associated with the cytochrome family, such as CYP2A7, CYP2C8, CYP2A6, CYP3A4, changed significantly, which was consistent with previous reports [27, 28]. However, few inflammatory cytokines or genes associated with cell growth changed significantly. Based on these results, host factors related to innate immunity may not sense HBV (at least under these replicating conditions), suggesting that this system may mimic the status of hepatitis B patients before seroconversion, in whom inflammation seldom occurs regardless of the high viral load.

Regarding changes in miRNA expression levels during HBV replication, among 2,019 mature miRNAs, 35 were upregulated and 14 downregulated by an increase or decrease of more than two-fold (Figure 1d and Supplementary Tables 3 and 4; complete datasets have been deposited as GEO accession number: GSE55929). Among these miRNAs, miR93-5p was significantly downregulated during HBV replication by more than 50%. Since miRNA93 regulates the expression levels of the MICA protein [23, 29], which is involved in the susceptibility to hepatocellular carcinoma in chronic hepatitis patients [24, 25], we focused on this miRNA in further analyses.

### Efficient delivery of miRNAs into liver cell lines using bionanocapsules

**Figure 2:**
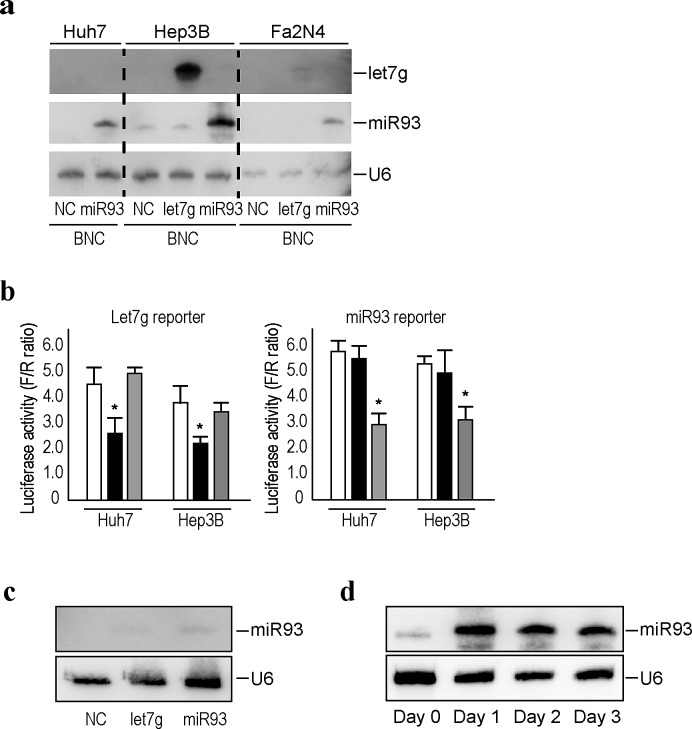
Efficient delivery of miRNAs into liver cell lines using BNCs a, Northern blotting of miRNAs delivered into liver cells by BNCs. Liver cancer cell lines, Huh7 and Hep3B, and primary hepatocytes immortalized by SV40, Fa2N4, were incubated with BNCs harboring the indicated miRNAs (miRNA93 or let7g) or BNCs without miRNAs (NC). After 24 hours, cells were harvested and subjected to analysis. Membranes were re-probed for let7g, miRNA93, and U6 as a loading control. The results shown are representative of three independent experiments. b, miRNAs delivered using BNCs were biologically functional. Huh7 and Hep3B cells were transfected with the indicated reporter constructs, which indicate the activity of each miRNA function. Twenty-four hours after transfection, cells were mixed with BNCs containing let7g (black bar), miRNA93 (gray bar), or negative control (white bar). Forty-eight hours after transfection, cells were subjected to a dual luciferase assay. Data shown represent the means ± s.d. of the raw ratios (FL/RL), obtained by dividing the firefly luciferase values by the renilla luciferase values, of three independent experiments. *p < 0.05. c, Delivery of miRNAs via BNCs were liver cell-specific. The 293T cells (human embryonic kidney cells) were incubated with BNCs containing let7g, miRNA93, or negative control (NC). After 24 hours, cells were subjected to Northern blotting for miRNA93. U6 was used as a loading control. The results shown are representative of two independent experiments. d, miRNA93 expression in Huh7 cells after the delivery of miRNA93 via BNCs. Cells were sequentially collected after incubation with BNCs containing miRNA93 and subjected to Northern blotting. U6 was used as a loading control. The results shown are representative of three independent experiments.

Efficient delivery methods of genes or compounds into targeted tissues or cells are essential to translate the *in vitro* results into clinical settings. Here, we utilized BNCs [21, 30, 31], which were originally developed to deliver genes and drugs with high efficiency and specificity to human liver-derived cells, as an efficient delivery method for miRNAs into human liver cells, including primary hepatocytes. Since BNCs are composed of HBV L proteins, the distribution of these BNCs and infected HBV should be similar. To confirm the efficiency of delivery of miRNAs into liver-derived cells by BNCs, we delivered BNCs carrying let-7g or miRNA93 to the human hepatocellular carcinoma cell lines, Huh7 and HepG2 cells, and to human normal hepatocytes immortalized with SV40 large T antigen, Fa2N4 cells [28]. The day after delivery of the BNCs, cells were collected and subjected to Northern blotting against let-7g, miRNA93, and U6, the loading control, and the results showed successful delivery of miRNAs into all cell lines tested (Figure 2a). The biological function of the delivered miRNAs was confirmed using luciferase-based reporters, which measured let-7g and miRNA93 functions [23]. Huh7 and Hep3B cells transfected with reporter constructs were delivered with let-7g or miRNA93 using BNCs, followed by a luciferase assay at the next day. Delivered miRNAs significantly decreased the corresponding luciferase activity, suggesting that the delivered miRNAs were functioning within the cells (Figure 2b).

We next examined the delivery of miRNAs into 293T cells (human embryonic kidney cell lines) to explore cell-specificity. Only a small increase in miRNA93 expression levels was observed 24 hours after transfer into 293T cells, based on Northern blots (Figure 2c), indicating that the BNCs had high specificity for hepatocyte-derived cells. The expression of transferred miRNA into Huh7 cells could be observed even 3 days after delivery (Figure 2d), suggesting that the delivered miRNAs are expressed for several days.

### miRNA delivery into human primary hepatocytes using bionanocapsules

**Figure 3:**
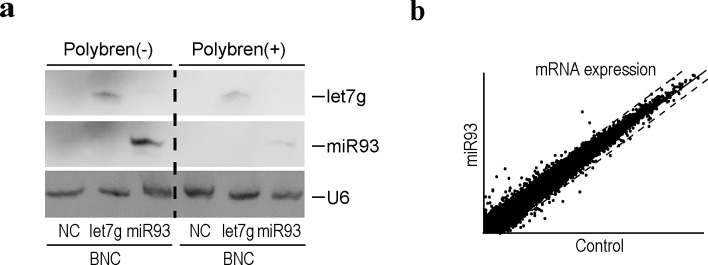
Efficient delivery of functional miRNAs into human primary hepatocytes using BNCs a, Northern blotting for miRNAs delivered into cells using BNCs. Human primary hepatocytes isolated from chimeric mice were incubated with BNCs containing the indicated miRNAs (miRNA93 or let7g) or BNCs without miRNAs (NC), with or without Polybren. After 24 hours, cells were harvested and subjected to analysis. Membranes were re-probed for let7g, miRNA93, and U6 as a loading control. The results shown are representative of three independent experiments. b, A scatter plot reflecting the transcriptome results between the control and primary human hepatocytes treated with BNCs containing miRNA93. Cells were harvested 24 hours after BNC treatment. Intensity normalization was performed using global normalization based on the expression levels of all genes analyzed. Dashed lines indicate the thresholds: a two-fold increase or 50% decrease in expression levels. The full data are deposited in GEO database accession: GSE55928.

Based on the efficient delivery of miRNA via BNCs into human liver-derived cell lines, we examined the BNC-mediated delivery of miRNAs into non-dividing human primary hepatocytes isolated from chimeric mice, as described above. BNCs could deliver miRNAs efficiently, even into non-dividing human primary hepatocytes, based on Northern blots (Figure 3a), irrespectively of the use of Polybren (Figure 3a).

Since the expression levels of miRNA93 were downregulated by HBV replication (Figure 1d and Supplementary Table 4), we delivered miRNA93 via BNCs into HBV replicating human hepatocytes to rescue the downregulation of miRNA93 levels and examine the effects of decreased miRNA93 on transcript levels (Figure 3b). The rescue of miRNA93 expression, recovered the baseline-level expression of some genes, such as 17-beta-hydroxysteroid dehydrogenase 14 (HSD17B14) and tripartite motif-containing protein 31 (TRIM 31), which were increased by HBV replication (Supplementary Table 1), suggesting that the mRNA levels of these genes may be directly or indirectly regulated by miRNA93. Although the enhanced decay of target transcripts by miRNAs has been reported [22, 32], miRNAs generally function as translational repressors [33]. However, these miRNA93 delivery results may not be accurate due to direct or indirect effects of miRNA93. In addition, changes in protein levels may differ from our transcript expression results.

### Modulation of MICA protein expression levels by delivery of miRNA93 using BNCs

**Figure 4:**
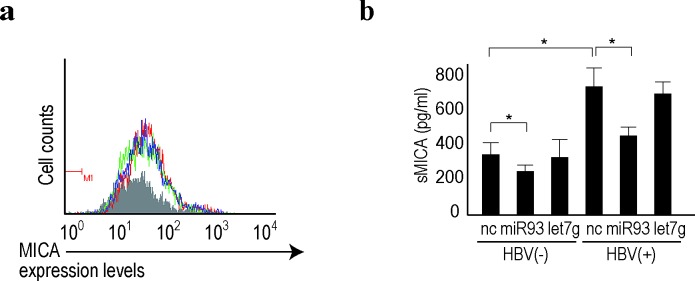
Soluble MICA protein levels were regulated by miRNA93 in human primary hepatocytes a, Membrane-bound MICA protein expression was not affected by miRNA delivery into human primary hepatocytes. Flow cytometric analysis of membrane-bound MICA protein expression in cells delivered BNC-mediated control (green line), let7g (blue line), or miRNA93 (red line). Gray-shaded histograms represent background staining, assessed using isotype IgG. Representative results from three independent experiments are shown. b, Soluble MICA protein levels in the supernatants of primary hepatocytes after delivery of the indicated miRNAs (let7g or miRNA93) or negative control (NC) with or without HBV replication. Data represent the means ± s.d. of three independent experiments. *p<0.05.

We previously identified miRNA93 as a critical regulator of MICA protein expression [23], which plays a role in the susceptibility to HBV-induced HCC [25]. MiRNA93 regulates MICA protein levels, but not transcript levels [23, 29]. Although it was found that miRNA93 expression levels decreased during HBV replication in primary hepatocytes (Figure 1d and Supplementary Table 4), MICA transcript levels were not affected (GEO accession number: GSE55928), suggesting that the effects of miRNA93 on MICA may be mediated by translational repression and not by mRNA decay, as we reported previously [23]. To confirm changes in the expression level of the MICA protein on the cell surface of primary hepatocytes induced by HBV infection, cells were subjected to FACS analyses. However, the protein expression levels on the cell surface did not change significantly (Figure 4a). MICA is a soluble protein released into the supernatant after shedding by ADAM10 and ADAM17[34]. Our results suggested that the modulated expression of MICA in primary hepatocytes during HBV replication affects this shedding process. To explore this possibility, we examined MICA protein levels in the supernatant using ELISA. As predicted, HBV infection significantly increased the protein concentration of MICA in the supernatant (Figure 4b).

Because an increase in soluble MICA levels in the serum of chronic hepatitis B patients is significantly associated with increased susceptibility to HCC [25], this increase during HBV replication needs to be prevented. Thus, we examined the effects of delivery of BNCs carrying miRNA93 into HBV-infected hepatocytes. Even though the MICA mRNA levels were not significantly affected by miRNA93 delivery based on microarray results (GEO accession: GSE55928), soluble MICA protein in the supernatant significantly decreased according to ELISA (Figure 4b). These results suggested that miRNA93 delivery into the liver decreases soluble MICA levels in the serum, which may be used to prevent HCC in chronic hepatitis B patients.

## DISCUSSION

We report that HBV replication in human hepatocytes decreases miRNA93 expression and increases soluble MICA levels. Increased soluble MICA levels in the serum are strongly associated with HBV-related HCC [25], and the increased soluble MICA levels could be antagonized by the delivery of miRNA93 into hepatocytes using BNCs. Thus, BNCs carrying miRNA93 may be used to prevent HCC in patients with chronic HBV infection.

Methods of efficient long-term HBV replication *in vitro* are not commonly available. Although transient transfection assays using fragments or tandem-units of the HBV genome or the full-length HBV genome without vector backbone have been applied [8-12], these models can be analyzed only for short-term replication after transfection. Although stable cell lines carrying HBV genomes are also used, HBV particles are derived from the HBV genome and integrate into the host genome, which differs from natural infection, in which HBV replication mainly relies on HBV cccDNA [6, 7]. Although the most ideal system for HBV infection and replication studies *in vitro* are primary human hepatocytes, they are difficult to obtain. Freshly isolated human hepatocytes from chimeric mice used in this report are relatively easily to obtain, since they proliferate under immune-deficient and liver-damaging conditions. These cells could support HBV replication for a substantial period and are valuable resources for studies on HBV infection and replication.

Another essential tool used in this study is that of BNCs. Primary hepatocytes are generally difficult to transduce with exogenous genes via transfection. Although viral-mediated gene transfer is useful even for primary cells, we chose BNCs as the miRNA delivery method for several reasons. First, since BNCs are composed of HBV L particles, these BNCs preferentially target primary hepatocytes and theoretically target similar cells as does HBV. Second, since we want to develop future therapeutics based on our experimental results, we avoided using viral materials such as lentiviruses or retroviruses to improve biosafety. Third, although BNCs have been established to transfer genes or drugs [21, 31, 35], transfer of miRNAs has not yet been examined, which prompted us to investigate delivery of miRNAs. We found that BNCs could efficiently deliver miRNAs into primary hepatocytes. Although further studies are required, delivery of miRNAs into hepatocytes via BNCs may be a promising approach to target hepatocytes *in vivo*, as BNCs are efficient delivery vehicles in xenograft models using human liver-derived cells [21].

The present results regarding comprehensive transcriptome analyses using HBV replicating hepatocytes may be applicable for future HBV research. While similar experiments are typically performed using transfection in HBV protein-expressing cells, or other relatively artificial experimental settings, the results here may better reflect the *in vivo* situation for HBV-infected hepatocytes. The expression of approximately 0.3% of genes changed during HBV replication when the threshold was set to a greater than 4-fold increase or to less than a 25% decrease. Although some of these genes were consistent with previous transcriptomic studies [36-38], we observed several novel characteristics. First, few inflammation-related genes were included among genes whose expression levels were significantly changed. The reason for this discrepancy remains unclear, but the results were considered accurate, since inflammation is rare when HBV replicates prior to seroconversion in chronic HBV-infected patients. Thus, HBV may be able to evade the sensing system related to innate immunity [39-41]. It should be explored whether changes in HBV sequences or the presence of host cells other than hepatocytes affect gene expression in hepatocytes *in vivo*. Second, based on comprehensive analysis of transcript changes, many CYP-related genes were upregulated during HBV replication, which is consistent with previous reports [27, 28]. Since the biological significance of these changes remain unclear, further studies are required to explore the biological significance during HBV replication.

Microarray analyses of changes in miRNA expression levels in HBV-replicating cells revealed that miRNA expression levels were not affected by HBV replication (2.4% among 2,000 miRNAs when the threshold was set to more than a two-fold increase or less than a 50% decrease). However, the miRNAs whose expression levels changed may play crucial roles in the regulation of target gene expression without affecting transcript expression levels, for example, targeting of the MICA protein by miRNA93, whose expression levels were downregulated by HBV replication. The results of comprehensive miRNA expression level analysis in HBV-replicating cells may increase our understanding of deregulated gene expression induced by HBV replication in hepatocytes.

MiRNA93 is a critical regulator of MICA protein expression [23, 29]. Thus, the decreased expression of miRNA93 by HBV suggested that the regulation of MICA expression by miRNA93 has biological significance. Polymorphisms in the MICA gene are associated with HBV and HCV-induced HCC [25, 42], and the increase in soluble MICA in the serum can be used as a susceptibility marker for HBV-induced HCC [25]. The increased levels of MICA protein expression agreed with the decreased miRNA93 expression. However, this increase was observed for soluble MICA protein levels and not membrane-bound MICA. While MICA is post-translationally dependent on the cell context or the status of viral infection [34], MICA may be readily processed from the cell surface in HBV-replicating primary hepatocytes and mainly released as soluble protein. Soluble MICA protein may function as a decoy for the NKG2D receptor in immune cells and as an evasion or immune surveillance system during chronic HBV infection. It may also be associated with HBV-induced HCC since HBV-infected hepatocytes may evade from the immune surveillance. Based on these results, BNCs carrying miRNA93 can be used to eliminate HBV-infected hepatocytes, which may be a novel approach for the prevention of subsequent virus-induced HCC.

## MATERIALS AND METHODS

### Cells

Primary human hepatocytes isolated fresh using the collagenase perfusion method from chimeric uPA/SCID mice with humanized livers [14, 17] were obtained from Phoenix Bio (Hiroshima, Japan). The purity of human hepatocytes was greater than 95%. A total of 3.0 × 10^5^ cells/well were seeded on a type I collagen coated-24-well plate and maintained in DMEM with 10% FBS, 5 ng/ml EGF, 0.25 μg/ml insulin, 0.1 mM ascorbic acid, and 2% DMSO [43]. These cells were able be maintained at a high density for more than 3 weeks, supporting the long-term replication of HBV infection *in vitro*.

### HBV infection *in vitro*

Serum from chronically HBV-infected patients with no HBe antibody before seroconversion was used for *in vitro* infection. Serum containing 9.0 log IU/ml of HBV genotype C in a volume of 3 μl, which is approximately 1.5 × 10^7^ copies of HBV, was added to the 3.0 × 10^5^ cells/well, followed by the addition of 4% PEG 8000 at day 0. Cells were washed, and the media was changed at days 1 and 2 and every 5 days thereafter. The media was collected to measure HBsAg and HBV-DNA at days 1, 2, 3, 7, 10, 15, 20 and 23 to confirm HBV replication. Measurements were performed at the clinical laboratory testing company SRL. Inc. (Tokyo, Japan).

### cDNA array and miRNA microarray

Human 25K cDNA microarray and human 2K miRNA microarray analyses were performed using miRNA oligo chips according to the standard protocols (Toray Industries, Tokyo, Japan). The data and the experimental conditions were deposited in a public database (GEO: accession numbers: GSE55928 and GSE55929).

### Bionanocapsules for miRNA delivery

Hollow particles consisting of HBV L proteins (pre-S1, pre-S2, and S regions) were used as the BNCs, as described previously [20, 21, 30]. The incorporation of miRNAs (miRNA93 or let-7g) into the hollow space and the delivery of miRNAs into human liver cells were performed as described previously [31]. Briefly, 32 μl BNC was added to 1 ml culture media at a final concentration of 50 nM miRNA 24 h before the indicated assays (unless otherwise specified).

### Northern blotting of miRNAs

Northern blotting of miRNAs was performed as described previously. Total RNA was extracted using TRIzol Reagent (Invitrogen, Carlsbad, CA, USA) according to the manufacturer's instructions. RNA (10 μg) was resolved on denaturing 15% polyacrylamide gels containing 7 M urea in 1× TBE and then transferred to a Hybond N+ membrane (GE Healthcare) in 0.25× TBE. Membranes were UV-crosslinked and prehybridized in hybridization buffer. Hybridization was performed overnight at 42°C in ULTRAhyb-Oligo Buffer (Ambion) containing a biotinylated probe specific for miRNA93 (CTA CCT GCA CGA ACA GCA CTT TG) and let-7g (AAC TGT ACA AAC TACT ACC TCA), which was heated to 95°C for 2 min prior to hybridization. Membranes were washed at 42°C in 2× SSC containing 0.1% SDS, and the bound probe was visualized using the BrightStar BioDetect Kit (Ambion). Blots were stripped by boiling in a 0.1% SDS, 5 mM EDTA solution for 10 min prior to rehybridization using a U6 probe (CAC GAA TTT GCG TGT CAT CCT T).

### Reporter plasmids, transient transfection, and dual luciferase assays

The firefly luciferase reporter plasmid was used to examine let7g and miRNA93 function. pGL4-TK, a renilla luciferase reporter, was used as an internal control [44]. Transfection and dual luciferase assays were performed as described previously [45].

### Flow cytometry

The expression levels of MICA on the cell surface were determined using flow cytometry, as described previously [23]. Briefly, cells were hybridized with anti-MICA (1:500; R&D Systems, Minneapolis, MN, USA) and isotype control IgG (1:500; R&D Systems) in 5% BSA/1% sodium azide/PBS for 1 h at 4°C. After washing, cells were incubated with goat anti-mouse Alexa 488 (1:1,000; Molecular Probes, Eugene, OR, USA) for 30 min. Flow cytometry was performed and the data analyzed using Guava Easy Cyte Plus (GE Healthcare, Little Chalfont, UK).

### ELISA for MICA

The concentration of MICA in the cell culture supernatant was measured using a sandwich ELISA, according to the manufacturer's instructions (R&D Systems, Minneapolis, MN, USA).

### Statistical analysis

Significant differences between groups were determined using the Student's *t*-test when variances were equal and using Welch's *t*-test when variances were unequal. *P*-values less than 0.05 were considered statistically significant.

## SUPPLEMENTARY MATERIAL AND TABLES


