# Supplementary material for: Specific delivery of microRNA93 into HBV-replicating hepatocytes downregulates protein expression of liver cancer susceptible gene MICA
Source: Oncotarget. 2014 Jun 26;5(14):5581–90. doi: 10.18632/oncotarget.2143 (PMC4170619; doi:10.18632/oncotarget.2143)
Supplement: Supplementary file 1 [file oncotarget-05-5581-s001.pdf]

Specific delivery of microRNA93 into HBV-replicating hepatocytes downregulates protein expression of liver cancer susceptible gene MICA

Supplementary Material

Supplementary Table 1: Genes upregulated by HBV infection in primary hepatocytes. Genes upregulated more than four-fold are indicated. The colored numbers indicate those genes with significant levels of expression (more than 10 in signal intensities and higher expression in darker color) and more reliable expression changes.

Gene Symbol	Gene description	Control	HBV	Ratio
CPSF6	Cleavage and polyadenylation specificity factor subunit 6	2	48	21.91
TDO2	Tryptophan 2,3-dioxygenase	82	1535	18.76
CYP2B7P1	CYP2B7P1 protein	2	33	17.02
HSD17B13	17-beta hydroxysteroid dehydrogenase 13 Precursor	9	125	13.19
HIST1H4I	Histone H4	2	23	12.82
APOA4	apolipoprotein A-IV precursor	952	12040	12.65
SLPI	Antileukoproteinase Precursor	99	1104	11.16
SHBG	Sex hormone-binding globulin Precursor	19	199	10.34
SAA1	Serum amyloid A protein Precursor	59	609	10.31
ZDHHC15	Palmitoyltransferase ZDHHC15	1	13	10.11
SLC3A1	Neutral and basic amino acid transport protein rBAT	7	66	9.76
KRT19	Keratin, type I cytoskeletal 19 (Cytokeratin-19)	4	37	9.01
HSD11B1	Corticosteroid 11-beta-dehydrogenase isozyme 1	11	100	9.01
CYP2A7	Cytochrome P450 2A7	280	2510	8.97
ANKRD33	Ankyrin repeat domain-containing protein 33	3	24	8.82
CIDEC	Cell death activator CIDE-3	5	40	8.27
MXRA7	Matrix-remodeling-associated protein 7 (Transmembrane anchor protein 1)	8	65	8.25
CYP2C8	Cytochrome P450 2C8	518	4010	7.74
HMGCS2	Hydroxymethylglutaryl-CoA synthase, mitochondrial Precursor (HMG-CoA synthase)	746	5615	7.52
SAA2	serum amyloid A2 isoform a	48	353	7.37
CYP2A6	Cytochrome P450 2A6	434	3127	7.21
TMEM63B	Transmembrane protein 63B	4	31	7.01

G0S2	Putative lymphocyte G0/G1 switch protein 2	68	475	6.98
STRADA	STE20-related adapter protein	112	753	6.72
CYP3A4	Cytochrome P450 3A4	296	1958	6.62
MAFF	Transcription factor MafF	37	243	6.57
APOA5	Apolipoprotein A-V Precursor	23	150	6.46
DHRS12	dehydrogenase/reductase	4	26	6.41
C10orf125	Protein fucU homolog	47	296	6.35
FGF21	Fibroblast growth factor 21 Precursor	27	172	6.31
CYP3A43	Cytochrome P450 3A43	3	20	5.90
NR1I3	Nuclear receptor subfamily 1 group I member 3	27	157	5.83
CCDC40	Coiled-coil domain-containing protein 40	2	10	5.70
INHBE	Inhibin beta E chain Precursor (Activin beta-E chain)	10	57	5.58
AGXT	Serine--pyruvate aminotransferase (SPT)	277	1539	5.55
L3MBTL	Lethal(3)malignant brain tumor-like protein	52	289	5.54
B3GNT3	UDP-GlcNAc:betaGal beta-1,3-N-acetylglucosaminyltransferase 3	6	31	5.45
RDH16	Retinol dehydrogenase 16	8	43	5.21
GPAM	Glycerol-3-phosphate acyltransferase 1	11	58	5.13
CYP3A4	Cytochrome P450 3A4	5204	2614 0	5.02
CYP4A11	Cytochrome P450 4A11 Precurso	59	290	4.91
AC011472.7	Hepatocellular carcinoma-associated protein TD26 Precursor	28	132	4.79
TXNIP	Thioredoxin-interacting protein	17	81	4.77
GPD1	Glycerol-3-phosphate dehydrogenase [NAD+], cytoplasmic	15	69	4.70
ADFP	Adipophilin (Adipose differentiation-related protein)	1966	9241	4.70
CREB3L3	Cyclic AMP-responsive element-binding protein 3-like protein 3	43	198	4.64
NOS3	Nitric oxide synthase, endothelia	9	41	4.64
PGLYRP2	N-acetylmuramoyl-L-alanine amidase Precursor	5	20	4.43
DOHH	Deoxyhypusine hydroxylase	230	1006	4.38
NR1I3	Nuclear receptor subfamily 1 group I member 3	43	185	4.34
S100P	Protein S100-P	10	43	4.34
CYP2D6	Cytochrome P450 2D6	74	310	4.21
ZG16	Zymogen granule membrane protein 16 Precursor	18	73	4.12
PRSS2	Trypsin-2 Precursor	41	168	4.08
ALDOB	Fructose-bisphosphate aldolase B	3659	1463 4	4.00

Supplementary Table 2: Genes downregulated by HBV infection in primary hepatocytes. Genes whose expression was downregulated by less than 25% are indicated. The colors used are defined in Supplementary Table 1.

Gene Symbol	Gene description	Control	HBV	Ratio
NPPB	Natriuretic peptides B Precursor	273	16	0.06
NCF2	Neutrophil cytosol factor 2	67	5	0.07
ACP1	Low molecular weight phosphotyrosine protein phosphatase (LMW-PTPase)	1021	85	0.08
MAP2	Microtubule-associated protein 2	10	1	0.12
TUBB3	Tubulin beta-3 chain	34	5	0.14
LOXL2	Lysyl oxidase homolog 2 Precursor	12	2	0.14
EDN1	Endothelin-1 Precursor	46	7	0.14
DCDC2	Doublecortin domain-containing protein 2	10	2	0.16
C5orf46	Uncharacterized protein C5orf46 Precursor	11	2	0.17
CXCL6	C-X-C motif chemokine 6 Precursor (Small-inducible cytokine B6)	31	5	0.17
TMEM27	Collectrin Precursor (Transmembrane protein 27)	140	25	0.18
TUBA1A	Tubulin alpha-1A chain	342	63	0.18
CCL2	C-C motif chemokine 2 Precursor (Small-inducible cytokine A2)	73	14	0.19
FILIP1L	Filamin A-interacting protein 1-like	78	16	0.20
XRCC4	DNA repair protein XRCC4	50	10	0.20
TAGLN	Transgelin (Smooth muscle protein 22-alpha)(SM22-alpha)	360	76	0.21
TNFRSF19	Tumor necrosis factor receptor superfamily member 19 Precursor	12	3	0.22
TPM2	Tropomyosin beta chain (Tropomyosin-2)(Beta-tropomyosin)	31	7	0.23
FGL1	Fibrinogen-like protein 1 Precursor	3452	835	0.24
DACT1	Dapper homolog 1	11	3	0.24
MYL9	Myosin regulatory light polypeptide 9	74	18	0.25

Supplementary Table 3: miRNAs upregulated by HBV infection in primary hepatocytes. The miRNAs whose expression was upregulated by more than two-fold are indicated. The orange-colored numbers indicate those genes with significant levels of expression (more than 100 in signal intensities).

microRNA	Control	HBV	Ratio
hsa-miR-4472	7.0	44.4	6.31
hsa-miR-4481	40.2	208.3	5.18
hsa-miR-660-5p	8.4	32.2	3.83
hsa-miR-124-3p	9.7	35.9	3.69
hsa-miR-4445-5p	9.2	33.5	3.63
hsa-miR-362-5p	11.4	41.2	3.60
hsa-miR-203a	7.9	25.9	3.27
hsa-miR-5698	13.5	43.0	3.19
hsa-miR-6127	11.1	35.0	3.16
hsa-miR-215	188.1	575.7	3.06
hsa-miR-4483	10.2	28.6	2.81
hsa-miR-335-5p	8.0	22.3	2.80
hsa-miR-3190-5p	6.9	18.7	2.69
hsa-miR-4676-5p	6.4	16.4	2.55
hsa-miR-935	7.2	18.2	2.53
hsa-miR-520a-3p	6.9	16.7	2.43
hsa-miR-3605-5p	7.2	17.2	2.41
hsa-miR-1273g-5p	9.6	22.3	2.31
hsa-miR-4456	9.0	20.2	2.25
hsa-miR-200a-3p	10.5	23.3	2.23
hsa-miR-212-3p	20.4	45.1	2.22
hsa-miR-3654	9.6	21.4	2.22
hsa-miR-194-3p	10.3	22.8	2.21
hsa-miR-3663-5p	27.9	61.5	2.20
hsa-miR-29c-3p	75.0	163.0	2.17
hsa-miR-4253	8.3	17.5	2.10

hsa-miR-222-3p	31.9	66.4	2.08
hsa-miR-378b	9.1	18.6	2.05
hsa-miR-5587-5p	8.2	16.9	2.05
hsa-miR-221-3p	91.1	186.0	2.04
hsa-miR-4700-3p	16.9	34.3	2.04
hsa-miR-29a-5p	6.5	13.0	2.01
hsa-miR-4716-3p	8.1	16.2	2.01
hsa-miR-5000-3p	9.5	19.0	2.00
hsa-miR-532-5p	13.3	26.6	2.00

Supplementary Table 4: miRNAs downregulated by HBV infection in primary hepatocytes. The miRNAs whose expression was downregulated more than 50% are indicated. The orange-colored numbers indicate those genes with significant levels of expression (more than 100 in signal intensities). mMRNA93-5p is indicated in pink.

microRNA	Control	HBV	Ratio
hsa-miR-424-5p	37.3	9.6	0.26
hsa-miR-5589-5p	51.6	15.2	0.30
hsa-miR-4470	23.8	7.5	0.31
hsa-miR-3620-3p	94.1	34.5	0.37
hsa-miR-1233-3p	50.4	20.2	0.40
hsa-miR-323a-5p	30.7	13.1	0.43
hsa-miR-6073	18.2	8.0	0.44
hsa-miR-365-3p	303.8	134.7	0.44
hsa-miR-4755-3p	67.5	30.4	0.45
hsa-miR-93-5p	559.0	258.5	0.46
hsa-miR-583	20.2	9.7	0.48
hsa-miR-143-3p	294.6	143.3	0.49
hsa-miR-3646	35.1	17.2	0.49
hsa-miR-4723-3p	15.3	7.6	0.50

Supplementary Table 5: Genes upregulated by HBV infection and rescued by BNC-delivered miRNA93. Those genes upregulated by HBV infection and subsequently downregulated by BNC-delivered miRNA93 in primary hepatocytes are indicated. The expression changes affected by BNCs containing miRNA93 alone are also provided. Colored numbers indicate those genes with significant levels of expression (more than 10 in signal intensities and higher expression in darker color).

Gene Symbol	Gene description	Control	HBV	Ratio	Control	miR93	Ratio	Control	HBV+miR93	Ratio
HSD17B14	17-beta-hydroxysteroid dehydrogenase 14	23	135	5.98	23	13	0.60	23	31	1.37
TRIM31	Tripartite motif-containing protein 31	9	43	4.62	9	8	0.82	9	13	1.43
MLC1	Membrane protein MLC1	4	16	4.34	4	3	0.93	4	4	1.04
RILP	Rab-interacting lysosomal protein	125	453	3.61	125	122	0.98	125	180	1.43
CSN1S1	Alpha-S1-casein Precursor	4	12	3.40	4	3	0.79	4	5	1.23
MT1A	Metallothionein-1A	362	1142	3.16	362	354	0.98	362	481	1.33
ZFP3	Zinc finger protein 3 homolog	4	13	3.10	4	4	0.88	4	5	1.29
ACOT2	Acyl-coenzyme A thioesterase 2	142	438	3.09	142	125	0.88	142	248	1.75
SAT2	Diamine acetyltransferase 2	473	1430	3.02	473	380	0.80	473	825	1.74
ARFGAP3	ADP-ribosylation factor GTPase-activating protein 3	164	479	2.92	164	137	0.84	164	284	1.73
AC005486.2	cDNA FLJ51355	19	52	2.67	19	19	0.99	19	28	1.46
SAT2	Diamine acetyltransferase 2	468	1216	2.60	468	452	0.97	468	885	1.89
AC074397.7	POM121-like protein DKFZp564N2472	63	160	2.56	63	47	0.75	63	92	1.46
LCE3B	Late cornified envelope protein 3B	3	7	2.55	3	2	0.89	3	4	1.58
ACADM	Medium-chain specific acyl-CoA dehydrogenase, mitochondrial Precursor	936	2268	2.42	936	841	0.90	936	1449	1.55
SLC24A4	Sodium/potassium/calcium exchanger 4 Precursor	3	8	2.42	3	2	0.59	3	2	0.53
CHRNA4	Neuronal acetylcholine receptor	3	6	2.37	3	2	0.91	3	3	1.31

	subunit alpha-4 Precursor									
ADH1C	Alcohol dehydrogenase 1A	8477	20034	2.36	8477	7608	0.90	8477	14295	1.69
AP001462.1	Putative uncharacterized protein	8	17	2.25	8	7	0.94	8	8	1.02
PPP4R4	Serine/threonine-protein phosphatase 4 regulatory subunit 4	11	25	2.24	11	10	0.94	11	14	1.26
CPT2	Carnitine O-palmitoyltransferase 2, mitochondrial Precursor	287	607	2.12	287	270	0.94	287	461	1.61
PUS1	tRNA pseudouridine synthase A	61	128	2.11	61	56	0.93	61	108	1.77
MAZ	Myc-associated zinc finger protein	2	5	2.11	2	2	0.84	2	1	0.64
MALL	MAL-like protein	9	18	2.10	9	9	0.99	9	17	1.99
